# Survival of people aged 50 years and older by HIV and HIV treatment status: findings from three waves of the SAGE-Wellbeing of Older People Study (SAGE-WOPS) in Uganda

**DOI:** 10.1186/s12981-020-00276-1

**Published:** 2020-05-14

**Authors:** Joseph Mugisha Okello, Stephen Nash, Paul Kowal, Nirmala Naidoo, Somnath Chatterji, Ties Boerma, Janet Seeley

**Affiliations:** 1 Medical Research Council/Uganda Virus Research Institute and London School of Hygiene and Tropical Medicine, Uganda Research Unit, P.O Box 49, Entebbe, Uganda; 2 MRC Tropical Epidemiology Group London School of Hygiene and Tropical Medicine, Keppel St, Bloomsbury, London, WC1E 7HT UK; 3 World Health Organization Division of Data, Analysis and Delivery for Impact, Avenue Appia 20, 1202 Geneva, Switzerland; 4 Chiang Mai University Research Center for Health Sciences, 110 Intavaroros Road, Sriphum, Muang Chiang Mai, 50200 Thailand; 5 Centre for Global Public Health, University of Manitoba, University of Manitoba, Winnipeg, MB R3T 2N2 Canada

## Abstract

**Background:**

Data on the survival status of older adults on antiretroviral treatment (ART) are scarce in sub-Saharan Africa. The objective of this study was to determine the survival status of people aged 50 years and older who were HIV-negative, HIV-positive not on ART, and HIV-positive on ART.

**Methods:**

We used three waves of data from the World Health Organisation Study on Global Ageing and adult health- Well Being of Older People Study cohort in Uganda, conducted in 2009, 2012–2013 and 2015–2016. The cohort included HIV-negative and HIV-positive persons aged 50 years and older recruited from multiple rural and peri-urban sites in Uganda. Data were collected using interviewer-administered questionnaire. Time-dependent ART data were collected from medical records using a data-abstraction form. This study was conducted before the universal test and treat policy came into effect. We fitted Cox survival models to estimate hazard ratios to compare the risk of death between groups, adjusted for age, sex, marital status and hypertension.

**Results:**

Of 623 participants, 517 (82.9%) of respondents had follow-up data and were included in this analysis. We observed 1571 person-years of follow-up from 274 people who were HIV-negative, and 1252 from 243 who were HIV-positive. The estimated mortality adjusted hazard ratio (aHR) was 1.89 (95% CI 1.0–3.4; p = 0.04) among people living with HIV compared to HIV-negative people. The aHR for mortality among people receiving ART compared with HIV-negative people was 1.75 (95% CI 0.9–3.5). People who were HIV-positive and not receiving ART had the greatest risk of death (aHR = 2.09, 95% CI 1.0–4.4 compared with HIV negative participants). The aHR for HIV-positive people not receiving ART, compared to those who were on treatment, was 1.19 (95% CI 0.6–2.5).

**Conclusion:**

Older adults living with HIV on ART had a risk of mortality that was nearly twice as high as HIV-negative adults. Further analyses of longitudinal data should be done to understand factors that affect the survival of older adults on ART.

## Background

The number of older people living with human immunodeficiency virus (HIV) is increasing globally [1]. Estimates from the Joint United Nations Programme on HIV/AIDS (UNAIDS) for 2013 indicated that around 3.6 million people aged 50 years and older were living with HIV. As of 2017, this estimate was 6.7 million older adults [1]. Even though HIV data availability for older people has improved, the 2018 UNAIDS estimates for those aged 50 years and older rely heavily on extrapolations of data for younger age groups [2]. Prior to 2010 global figures were found to underestimate the total number of people aged 50 years and older living with HIV globally [3]. After 2010, UNAIDS changed its models for estimating the number of older adults living with HIV. There may still be underestimates but we do not have consistent data on this. Results from a study following cohorts between 1998 and 2011 in Zimbabwe showed that HIV prevalence among those aged 45–54 years was higher than in those aged 15–44 years between 2006/08 and 2009/11 [4].

In 2005 in the USA, 15% of the new HIV diagnoses and 24% of those living with HIV were aged 50 years and over. In 2015, 17% of new diagnoses and 47% of the people living with HIV in the USA were aged 50 years and older [5]. In sub-Saharan Africa (SSA) where the largest numbers of people living with HIV live, the number of people aged 50 years and older living with HIV has continued to increase. In 2017, it was estimated that four million people aged 50 years and over were living with HIV in SSA [1].With the increasing number of people accessing combination antiretroviral therapy (ART) in Africa, these numbers are likely to be higher currently.

A number of factors contribute to the increase in the number of older people living with HIV, including the introduction of effective HIV treatment with ART which has significantly prolonged the life expectancy of people on ART treatment [6], and older people acquiring new HIV infections, especially in SSA where HIV prevention campaigns do not target older adults.

An estimated 59% of people living with HIV in 2017 were on ART [1]. ART prolongs the life expectancy of people living with HIV, and as a result of this, populations taking ART are living longer now than when antiretroviral drugs were introduced in Africa in early 2000. Data are scarce on how the natural decline in immune function with age might influence outcomes in older people living with HIV. People aged 50 and older on ART have good immunological and virological responses [7–10], and report better adherence to treatment than younger adults [11–13] but studies have reported higher rates of disease progression, shorter survival times and higher rates of progression to AIDS in older than younger adults [14–17]. This has been attributed to late diagnosis and treatment of older people living with HIV in addition to higher competing mortality risks [8]. In addition, the natural decline of immune function with ageing might influence mortality among those newly diagnosed with HIV when they are aged 50 years and over as compared with those diagnosed younger, who maintain viral load suppression on ART for decades through to older age.

ART has increased life expectancy for those on treatment, and has also improved the health and functioning of older adults living with HIV on ART. Studies following older people with and without HIV in both Uganda [18, 19] and South Africa [20] have demonstrated that older people with HIV on ART report the same or even better health as compared to their counterparts who are HIV-negative. Most of these studies have been cross-sectional studies and have been conducted in high-income countries, leaving an information gap on survival of older people on ART in the African region. The objective of this paper was to document the survival rates of people aged 50+ years by HIV and treatment status.

### Data and methods

#### SAGE-WOPS HIV study

The SAGE-WOPS (Study on global AGEing and adult health –Wellbeing of Older People Study) HIV study is a cohort study of people aged 50+ years established in Uganda in 2009 [18, 21], as a collaboration between WHO and the Medical Research Council/Uganda Virus Research Institute and London School of Hygiene and Tropical Medicine Uganda Research Unit. Three survey waves were conducted within the cohort.

SAGE-WOPS wave 1 was conducted in 2009–2010 [18] using a random-selection of the surveillance population. The surveillance population included the General Population Cohort [22–24] and the Entebbe cohort [25–27]. Initial recruitment into the cohort was in 2009 in two districts (Kalungu and Masaka) in Southwest Uganda and a third (Wakiso) near Entebbe town on the shores of Lake Victoria. The initial study population consisted of 510 participants aged 50 years and over who were living with and without HIV. The study population included both those on treatment with ART and those waiting to start ART according to the government of Uganda ART treatment guidelines at that time (WHO stage IV disease irrespective of CD4 cell count, WHO stage III disease if CD4 cell test testing was unavailable; if available CD4 cell counts ≤ 200 cells/mm^3^. Other criteria were CD4 cell counts above 200 but below 350 cells/mm^3^ in those co-infected with pulmonary tuberculosis or have severe bacterial infection and women who were pregnant. The last criterion was WHO stage 1 and 2 with CD4 cell counts ≤ 200 cells/mm^3^. HIV-positive people and HIV-negative people were selected from the same study settings and cohorts.

During SAGE-WOPS wave 2 (2012–2013), we visited households of wave 1 participants. If participants were still living in the study area, we re-interviewed them. If the respondent was not available, we obtained information about the survival status. Of the 510 wave 1 participants, 63 (12.4%) had died and 148 (29.0%) were not seen during wave 2 [28]. In wave 2, we recruited an additional 100 older people living with HIV attending The AIDS Support Organisation (TASO) that provides care to people living with HIV in Masaka town. The new participants were randomly selected from people aged 50 years or older attending TASO. These additional recruits were to increase the number of people living with HIV in the cohort. In order to avoid misclassification of the study groups, all older people who were HIV-negative in SAGE-WOPS1 were retested for HIV using the Uganda Ministry of Health algorithms for rapid HIV testing. Study participants were provided pre- and post-test counselling in accordance with the Uganda Ministry of Health HIV testing policy. Those found positive were referred to both private and public health care facilities in the study setting that are accredited with the Ministry of Health in Uganda to provide ART to people with HIV.

In SAGE-WOPS wave 3 (2015–2016), all participants in waves 1 or 2 who were still living in the study area were tracked and re-interviewed. In addition, all people who had become aged 50 years or older who were living with HIV within one of the MRC/UVRI and LSHTM HIV surveillance sites in Southwest Uganda (Kalungu) were eligible for the cohort and interviewed.

In all 3 survey rounds, data on demographic characteristics of study participants were collected. In addition, during SAGE-WOPS wave 3, data on survival status for all participants who were seen in the previous SAGE-WOPS waves were collected.

For participants who died between the baseline survey and the follow-up surveys, mortality data were collected using a standardized verbal autopsy questionnaire adapted from the World Health Organization verbal autopsy questionnaire [29]. The resulting data included the date of death and the suspected cause of death. We obtained notification of deaths from the next of kin or any other close relative of the deceased.

ART-related data were collected through review of clinical files of participants who were on ART treatment and time-updated ART status was used in the analysis. These were available only for study respondents recruited from Kalungu district (about half of the participants on ART). It was not possible for the study team to review files for participants recruited from Wakiso district because they were receiving ART treatment from multiple health care facilities and their ART records were incomplete and diverse.

*Hypertension status* For all study participants (all three waves), systolic and diastolic blood pressures were measured three times at recruitment in WOPS with participants in a sitting position using a Boso Medistar-S-wrist blood pressure monitor. An average BP for the three readings was computed and used in the analysis. Hypertension was defined according to the World Health Organization criteria (systolic blood pressure ≥ 140 mmHg and/or diastolic blood pressure ≥ 90 mmHg).

#### HIV testing

At each study wave, follow-up participants who were HIV-negative in the previous wave were retested in order to avoid misclassification. HIV testing was done following the Ministry of Health of Uganda testing algorithm. The algorithm for HIV rapid testing consisted of an initial screening with the rapid test, Determine HIV1/2. If the test result was negative, the participant was given a negative diagnosis with no further rapid testing. If the test result was positive, the sample was retested with the rapid test HIV 1/2 Stat-Pak. If both tests gave a positive result, the participant was given a diagnosis of HIV-positive with no further rapid testing. If the tests gave discordant results (i.e. one positive and the other negative), the sample was further evaluated with the rapid test Uni-Gold Recombinant HIV-1/2. For those samples assessed by all three tests, two positive test results were interpreted as a positive diagnosis. If two of the three tests gave negative results, then the participant was considered to be HIV-negative.

#### Ethical issues

We obtained ethical clearance for all SAGE-WOPS waves from the Uganda Virus Research Institute Research and Ethics Committee and from the Uganda National Council for Science and Technology. Approval was also obtained from the WHO Ethical Review Committee (RPC-149). All study respondents gave a written/thumb printed consent after the study interviewers read for them an information sheet with details about the study.

### Statistical methods

In this paper, the study population was restricted to those who were present in Wave 1 or Wave 2 and had at least one follow-up visit, or who died during the follow-up period. The outcome of interest was overall survival, specifically investigating two binary time-updated exposures: HIV status and ART status (among those who were HIV-positive). Primary analysis was performed using time-to-event methods: entry was the date of interview in Wave 1, and exit was either date of death or date last seen (date of last SAGE-WOPS interview).

The initial analysis was descriptive, including Kaplan–Meier plots, number of deaths tabulations, and the person-years at risk for each group. To assess the impact of HIV on overall survival, we fitted a Cox survival model, thus estimating a hazard ratio (HR) to compare the risk of death between groups. We assessed the impact of ART treatment by including a three-level indicator variable to indicate if a person was HIV-negative, HIV-positive and on ART or HIV-positive and not on ART. We controlled for age by using date of birth as the origin in the model. HIV sero-status and ART treatment status were recorded at each wave and included in the analysis as time-varying covariates. We adjusted for key confounders (sex, marital status (currently married or not) and hypertension) as well as current age. Due to sparse data, we could not include further covariates in the model, judged by the rule of thumb of ten events per parameter. As an exploratory investigation, we examined if the effect of HIV differed by sex by including an interaction between HIV and sex in the regression model.

We carried out two further analyses. First, we examined the effect of ART treatment in a dataset restricted to just people living with HIV. Second, to confirm the results from the main analysis we fitted an exponential survival model, split into ten-year age bands to compare people of similar ages and to estimate adjusted risk ratios (RR) for the two main exposures listed above. All estimates are reported with a 95% confidence interval (CI). Stata version 14.2 (Arizona, USA) was used for all analyses.

## Results

**Fig. 1:**
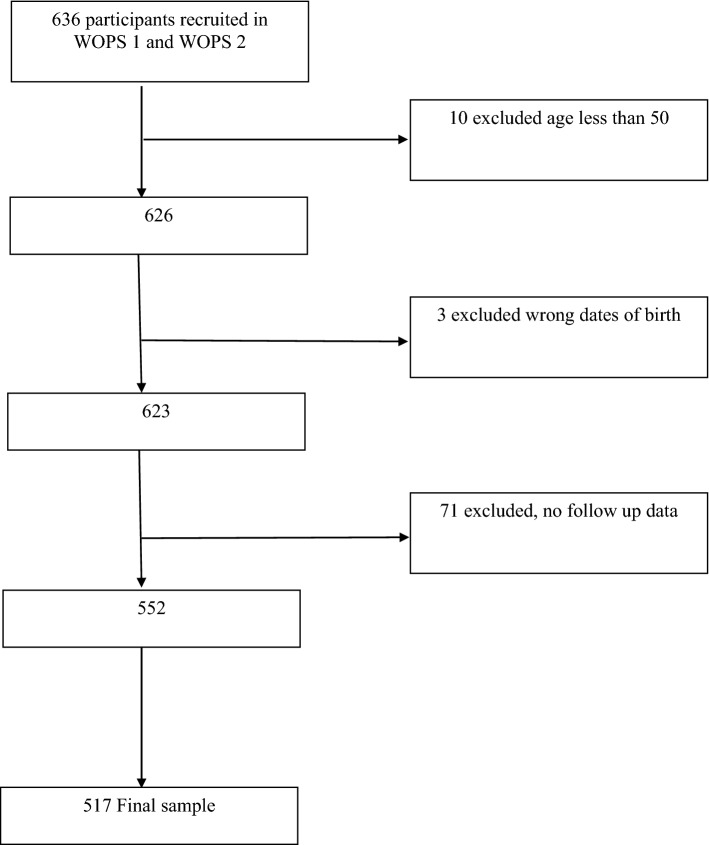
Flow of participants

In SAGE-WOPS wave 1, a total of 510 people was recruited; a further 126 were recruited for wave 2, giving a total of 636 potentially eligible for this study. Of these, 10 were ineligible as they were younger than 50 at recruitment and 3 had a reported date of death through surveillance earlier than recruitment. Of the remaining 623 participants, 71 did not have follow-up data and were not reported as having died, so were excluded from the analysis, and 35 had missing data and were excluded from the complete case analysis (8 due to missing data on sex, 27 with missing date of birth). For flow of participants, refer to Fig. 1.

Of the 517 respondents included in this analysis, the majority were women (61%); the median age at entry was 61 years (Table 1). Participants who were lost to follow-up were less likely to be married and more likely to be hypertensive, compared to people included in this analysis (Table 1).

**Table 1:** Baseline characteristics of study participants and those who were lost to follow-up

Characteristic	HIV-negative	HIV-positive	LTFU	
			HIV-negative	HIV-positive
N (%)	274 (100%)	243 (100%)	36 (14.6%)	35 (14.4%)
Wave 1	266 (97.1%)	169 (69.5%)	34	21
Wave 2	214 (78.1%)	201 (82.7%)	2	14
Wave 3	205 (74.8%)	200 (82.3%)	–	–
Female sex	98 (35.8%)	102 (42.0%)	12 (16.9%)	16 (22.5%)
Married	95 (34.7%)	79 (32.5%)	7 (9.9%)	10 (14.1%)
Hypertensive	51 (18.6%)	20 (8.2%)	14 (19.7%)	4 (5.6%)
ART	0 (%)	155 (63.8%)	0 (0.0%)	26 (74.3%)
Age at entry; median (IQR)	69.2 (60–77)	56.6 (53–62)	69 (60–78)	57 (52–63)

In this analysis, we included 243 people who were living with HIV; just one person seroconverted during the study period, between Wave 2 and Wave 3. The proportion of people living with HIV who were on ART increased from 50.9% (86/169) at wave 1 to 78.1% (157/201) at wave 2, and 72.2% (143/198) at wave 3.

We observed a total of 2823 person-years of follow-up; 1571 from people who were HIV-negative, and 1252 from those were HIV-positive. HIV negative respondents were significantly older than HIV positive participants at enrolment (median age 69.2 years vs 56.6 years; p < 0.0001). HIV negative participants were also less likely to be female and more likely to be hypertensive, and all analyses are adjusted for age, sex and hypertension. In addition, loss to follow-up was differentially associated with marital status by HIV status and this was adjusted for.

### Overall survival by HIV status

We were notified of 74 (14.3%) deaths among the 517 study participants. Of these, 45 were among people who were HIV-negative (16.9% of participants who were HIV-negative at baseline) and 29 were among people living with HIV at baseline (15.4%). The mortality rate was 28.6 (95% CI 21.4–38.4) per 1000 person-years among HIV-negative participants, and 23.2 (95% CI 16.1–32.3) among people living with HIV, reflecting the older age at enrolment of HIV negative participants. The median age of death in the two groups was 85 and 77 years, respectively. After adjusting for age, marital status and hypertension, there was evidence of poorer survival among HIV-positive than HIV-negative respondents (p = 0.02; Fig. 1), with an estimated adjusted HR of 1.89 (95% CI 1.0–3.4; Table 2). There was no evidence of effect-modification of this association by sex (p = 0.68). The estimated adjusted rate ratio from the exponential survival model was similar (aRR = 1.70; 95% CI 1.0–3.0; p = 0.07).

**Table 2:** Results from Cox model for risk of death by HIV status

N = 517	Adjusted hazard ratio^a^	95% CI	p value
HIV negative	1		
HIV-positive	1.89	1.03–3.45	0.04
Female sex	0.57	0.33–0.98	0.04
Married	0.61	0.33–1.13	0.12
Hypertensive	1.33	0.71–2.48	0.38

^a^Adjusted for age, sex, marital status and hypertension

HIV-positive participants not receiving ART had the greatest relative risk of death (aHR 2.09, 95% CI 1.0–4.4, p = 0.05, compared with HIV negative participants; Table 3). HIV-positive participants on ART also had a higher relative risk of death than HIV-negative people (aHR = 1.75, 95% CI 0.9–3.5, p = 0.11; Table 3), although this was not statistically significant. The aHR for HIV-positive people not receiving ART, compared to those on ART was 1.19 (95% CI 0.6–2.5). The adjusted RR from an exponential model were similar.

**Table 3:** Results from Cox model for risk of death by ART status

N = 517	Hazard ratio	95% CI	p value
HIV negative	1		
HIV-positive, on ART	1.75	0.88–3.47	0.11
HIV-positive, not on ART	2.09	1.00–4.37	0.05
Female sex	0.57	0.33–0.97	0.04
Married	0.61	0.33–1.12	0.11
Hypertensive	1.33	0.71–2.49	0.37

### Overall survival by ART status

**Fig. 2:**
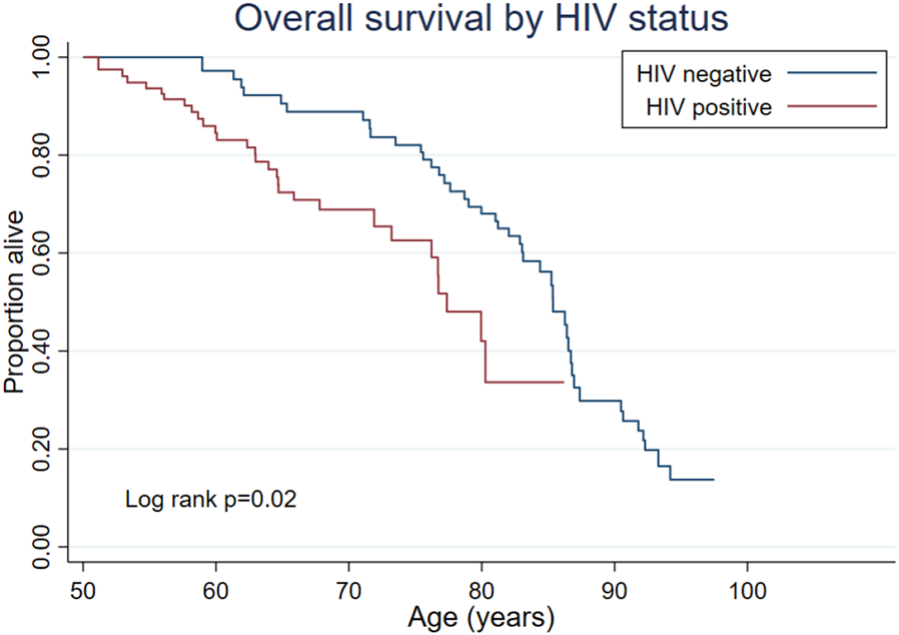
Overall survival by HIV status

Among the 243 people living with HIV, we observed 29 deaths; 12 were among people not receiving ART at the visit prior to their death (from 449 person-years of observation), and 17 were respondents who reported using ART at their last visit to the health facility (802 person-years). The rate of death was 26.7 (95% CI 15–47; median age of death 77 years) per 1000 person years and 21.2 (95% CI 13–34; median age of death 77 years) among the two groups respectively. A log-rank test comparing the two groups found no evidence of a difference in survival (p = 0.85), although we note the small number of deaths would reduce power to detect any difference here. A Kaplan–Meier plot of survival times is shown in Fig. 2 (Kaplan–Meier plot of overall survival, by HIV treatment status). The estimated aHR was 0.85 (95% CI 0.4–1.8; p = 0.67). There was no evidence (p = 0.45) of a differing effect of ART between men and women. The estimated aRR from an exponential model was similar (aRR = 0.79; 95% CI 0.4–1.7).

## Discussion

In this study, we explored survival by HIV and HIV treatment status in people aged 50 years and over. After adjusting for age and other key confounders, participants aged 50 years and over who were HIV-negative had better survival than people aged 50 years and over who were HIV-positive and were on ART or were still waiting to initiate ART. However, among HIV positive participants, there was no evidence of a difference in survival by ART status.

From the previous cross-sectional analyses from WOPS, we have consistently established that older people who are living with HIV reported similar or better health status compared to those who are HIV-negative of the same age [18, 30]. The possible explanation for this has been the frequent interaction of HIV-positive older persons with the health-care facilities as opposed to those who are HIV-negative who may take not visit health care facilities regularly [31].

**Fig. 3:**
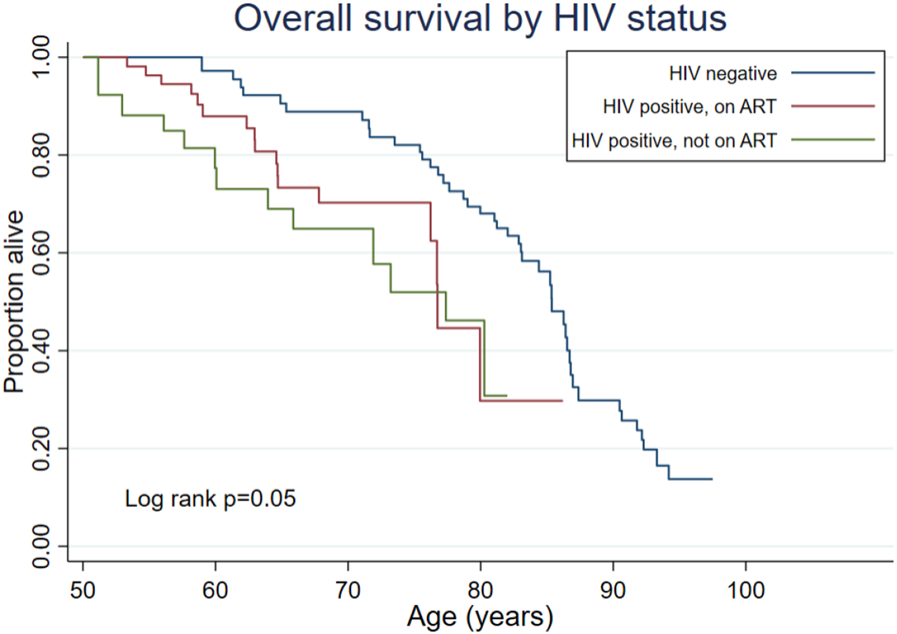
Overall survival by ART status

Older people who had not yet initiated ART had similar survival with those who were already on ART. One possible explanation for this could be the fact that we were unable to adjust for baseline CD4 cell counts: these data on baseline CD4 cell counts were not available for most of our study participants due to the fact that by 2004 when ART was introduced in the study population patients could be started on ART based on other criteria, irrespective of if they had a known baseline CD4 cell count. It should however be noted that all the participants who were on ART had been on ART for 6 months or more and those who had not yet started ART were still healthy without any documented opportunistic disease. Some of the factors that may influence survival of older patients on ART include the time of HIV diagnosis, the period the patient has been on treatment, availability of opportunistic infections and more importantly adherence to antiretroviral medication. However, most of these variables were not related to survival in this paper. Other factors include co-existence of multimorbidity in patients with HIV infection (Fig. 3).

There are limited longitudinal data within SSA which cover the health and survival of older people living with HIV and on treatment with ART. Some of the few longitudinal studies that have been done only compared response to ART treatment and survival between older adults (50 years and above) and younger adults (those aged below 50) [32]. In addition, most of these studies have not compared the survival of older adults who are HIV-negative to those living with HIV. This study contributes to the scanty literature available on survival of HIV infected older adults within the African region.

There are some limitations to our study. First, we collected ART related data by reviewing ART related records only from study participants on ART from Kalungu district. It was not possible for the study team to review files for participants recruited from Wakiso district because they were receiving ART treatment from multiple health care facilities and their ART records were incomplete and diverse. This could have introduced some bias in our analyses. However, since participants from Kalungu represent about half of all WOPS participants on ART, the bias would be minimised. Second, we intended to collect adherence-related data from all our study participants but this was not possible because these adherence data are not routinely collected within the facilities where our study participants obtain their antiretroviral drugs. In addition, ART treatment status was recorded at each wave, and included in analysis as a time-varying variable. As such, we did not know the actual date people started ART or stopped ART. Age reporting may be problematic, as older persons often do not know their exact age and they do not have birth certificates. Therefore, errors in age is possible. In our cohort studies, major efforts were made to collect the best possible data on age. Most old people in Uganda know major events that occurred around the time of their birth. We asked for major events and used the dates of these events to estimate their age. Date of death through surveillance may also not be very accurate as exemplified by 3 participants that were excluded because they had reported date of death earlier than recruited.

These data were collected before the universal test and treat policy was put in place in Uganda. When SAGE-WOPS cohort was initiated, those eligible for ART initiation were those in WHO clinical stage 3 or 4, or those who had a CD4 cell count of less than 200/µL. As the cohort enrols for the fourth wave, all the study participants who were originally HIV-positive have already been initiated on ART following the implementation of test and treat policy by WHO [33], which has been adopted by the Uganda Ministry of Health.

## Conclusion

Our study found that older adults living with HIV, including those on ART had a risk of mortality that was nearly twice as high as HIV-negative older adults. Further analyses of longitudinal data should be done to understand factors that affect the survival of older adults on ART.

## Data Availability

The datasets analysed during the current study are available on the WHO website on Global Ageing and Adult Health (SAGE), SAGE-WOPS HIV studies https://www.who.int/healthinfo/sage/hiv_studies/en/.

## Abbreviations

SAGE: World Health Organisation study on Global Ageing and Adult Health
WOPS: Wellbeing of Older People Study
ART: Antiretroviral Therapy
SSA: Sub Saharan Africa
MRC: Medical Research Council
UVRI: Uganda Virus Research Institute
LSHTM: London School of Hygiene and Tropical Medicine
